# Decompressive hemicraniectomy as a salvage therapy in the Neuro-ICU: a meta-analysis of neurologic outcomes for malignant middle cerebral artery infarction

**DOI:** 10.3389/fmed.2026.1843890

**Published:** 2026-07-14

**Authors:** Jiatong Hu, Qi Yang, Zhuqing Li

**Affiliations:** 1 Department of Acupuncture and Moxibustion Ward (Neurology), Guang’anmen Hospital, China Academy of Chinese Medical Sciences,, Beijing, China; 2 Department of Gynecology, Xiyuan Hospital, China Academy of Chinese Medical Sciences, Beijing, China; 3 School of Chinese Medicine, Nanjing University of Chinese Medicine, Nanjing, China

**Keywords:** decompressive hemicraniectomy, functional outcome, malignant middle cerebral artery infarction, meta-analysis, neurocritical care

## Abstract

**Background:**

Malignant middle cerebral artery infarction is a devastating condition associated with high mortality and poor functional outcomes despite maximal medical management. Decompressive hemicraniectomy has emerged as a salvage therapy to reduce intracranial pressure and prevent cerebral herniation, but uncertainty remains regarding functional outcomes across different follow-up periods and patient populations.

**Methods:**

We conducted a systematic review and meta-analysis of randomized controlled trials evaluating decompressive hemicraniectomy versus medical management in patients with malignant middle cerebral artery infarction. A comprehensive search of PubMed, Embase, and the Cochrane Central Register of Controlled Trials was performed from inception through 18th February 2026. The primary outcomes were mortality and favorable functional outcome, defined as modified Rankin Scale score of 0 to 4. Secondary outcomes included survival with severe disability, National Institutes of Health Stroke Scale scores, and Barthel Index scores. Pooled effect estimates were calculated using random-effects models. Heterogeneity was assessed using the I^2^ statistic.

**Results:**

Fourteen randomized controlled trials comprising 1,003 patients were included. Decompressive hemicraniectomy significantly reduced mortality at 30 days (risk ratio: 0.26, 95% confidence interval: 0.16 to 0.50), 6 months (risk ratio: 0.43, 95% confidence interval: 0.12 to 0.57), and 12 months (risk ratio: 0.46, 95% confidence interval: 0.13 to 0.59), with sustained benefit at 36 months. Favorable functional outcome was significantly improved at 3 months (risk ratio: 1.86, 95% confidence interval: 1.31 to 2.63) and 6 months (risk ratio: 1.58, 95% confidence interval: 0.94 to 2.67), but not at 12 months. Survival with severe disability did not differ significantly between groups at either 6 or 12 months. Barthel Index scores showed significant improvement favoring surgery at 3 and 6 months, though substantial heterogeneity was observed. Long-term follow-up demonstrated significant improvements in National Institutes of Health Stroke Scale and Barthel Index scores favoring the surgical group.

**Conclusion:**

This updated meta-analysis of 14 RCTs (1,003 patients) provides three novel insights beyond prior syntheses. First, the mortality benefit of decompressive hemicraniectomy is sustained across all ages and time points, consistent with prior reports. Second, age-stratified analysis reveals that older patients (≥60 years) derive similar survival benefits but significantly poorer functional outcomes compared with younger patients. Third, time-dependent analysis demonstrates that functional benefits are significant at 3 and 6 months but not at 12 months, a trajectory not previously characterized. These findings refine the risk–benefit calculus for shared decision-making in the Neuro-ICU, particularly for older patients and for expectations regarding long-term functional independence.

## 1 Introduction

Malignant middle cerebral artery infarction represents one of the most formidable challenges encountered in the neurocritical care environment. Constituting approximately 10 to 15 percent of all supratentorial ischemic strokes, this devastating condition is defined not merely by the initial ischemic insult but by its catastrophic natural history (1). The term malignant was first coined by Hacke and colleagues in 1996 to describe a specific subtype of large hemispheric infarction characterized by a fulminant, often fatal, clinical course driven by progressive cerebral edema. The pathophysiological sequence is relentless: following the occlusion of the distal internal carotid artery or the proximal middle cerebral artery, extensive ischemic necrosis occurs, affecting not only the cortex but also the underlying white matter and basal ganglia (2). Within the first 24 to 72 h, cytotoxic edema transitions to vasogenic edema, leading to a rapid increase in intracranial volume (3). As the rigid confines of the cranium prevent expansion, intracranial pressure rises exponentially. This leads to the displacement of midline structures, compression of the contralateral ventricle, and ultimately, transtentorial herniation. Historically, despite maximal medical management, the mortality rate associated with malignant middle cerebral artery infarction approached 80 percent, with most deaths resulting from cerebral herniation within the first week of ictus. Those few patients who survived often did so with profound, disabling neurological deficits, underscoring the dire prognosis that has long defined this condition (4).

The cornerstone of initial management in the Neuro-ICU revolves around medical therapies aimed at mitigating cerebral edema and controlling intracranial pressure. Standard protocols include head-of-bed elevation, osmotic therapy with mannitol or hypertonic saline, sedation, and in some cases, hypothermia. However, it has become increasingly evident over the past three decades that these medical interventions are largely temporizing measures (5). They address the physiological consequences of edema without altering the fundamental anatomical constraint that prevents the swollen brain from expanding. The limitations of medical management are stark: while these therapies may transiently reduce intracranial pressure, they do not prevent the delayed, irreversible herniation that constitutes the primary mechanism of mortality in this population. This therapeutic ceiling has driven the search for a more definitive, mechanically effective salvage strategy, leading to the introduction of decompressive hemicraniectomy (6).

Decompressive hemicraniectomy is a surgical procedure that involves the removal of a large segment of the skull, typically a frontotemporoparietal bone flap, with a concurrent duroplasty, thereby converting the rigid cranial vault into a compliant, open chamber (7). This intervention does not reverse the ischemic injury itself, but it fundamentally alters the intracranial pressure-volume relationship. By providing ample space for the edematous brain to expand outward, decompressive hemicraniectomy prevents the downward and medial shifts that precipitate brainstem compression (8). In doing so, it preserves cerebral perfusion pressure, reduces the risk of secondary ischemic injury to penumbral tissue, and most importantly, interrupts the cascade toward herniation (9). As a salvage therapy, decompressive hemicraniectomy is typically deployed when the clinical trajectory suggests failure of medical management, often serving as a final intervention to avert mortality. However, the ethical and clinical complexity of this procedure lies in its dual-edged nature: while it is extraordinarily effective at preserving life, the quality of that life remains highly variable, ranging from functional independence to a state of severe disability necessitating institutional care (10).

The contemporary evidence base for decompressive hemicraniectomy in malignant middle cerebral artery infarction is largely derived from a series of landmark randomized controlled trials conducted in Europe between the late 1990s and the mid-2000s. The three most influential trials, known as the Decompressive Surgery for the Treatment of Malignant Infarction of the Middle Cerebral Artery trial, the Decompressive craniectomy in Malignant Middle Cerebral Artery Infarction trial, and the hemicraniectomy after Middle Cerebral Artery Infarction with Life-threatening Edema Trial, collectively established the efficacy of surgery in reducing mortality (11). When these trials were pooled in prior meta-analyses, the results were unequivocal: decompressive hemicraniectomy significantly reduces the risk of death compared to conservative management. Specifically, the number needed to treat to prevent one death was strikingly low, often ranging between two and four patients. This compelling survival benefit has cemented decompressive hemicraniectomy as the standard of care for patients with malignant hemispheric infarction who meet specific clinical and radiographic criteria (12).

Despite the clear mortality benefit, the interpretation of functional outcomes remains a subject of intense debate and clinical nuance. The primary functional outcome measure in these studies has been the modified Rankin Scale, a six-point disability scale ranging from zero, representing no symptoms, to six, representing death. Historically, a favorable outcome in these trials was defined as a modified Rankin Scale score of zero to three, which includes patients with moderate disability who are able to walk without assistance (13). This distinction becomes critically important when examining the divergent outcomes observed across different age groups.

Age has emerged as the single most important moderator of treatment effect in the context of decompressive hemicraniectomy (14). The original European trials predominantly enrolled patients under the age of 60, demonstrating not only a reduction in mortality but also a reasonable probability of achieving functional independence. However, the incidence of malignant middle cerebral artery infarction increases with age, and the aging population necessitates a clear understanding of surgical benefit in older adults. The subsequent DESTINY II trial specifically addressed this gap by randomizing patients aged 61 years and older. This finding ignited a profound ethical discussion regarding the goals of care in the Neuro-ICU, forcing clinicians to weigh the preservation of life against the potential for a survival outcome that may be perceived by some patients and families as unacceptable (15).

Beyond age, several other factors contribute to the complexity of synthesizing the evidence on decompressive hemicraniectomy. There is substantial heterogeneity in the timing of surgical intervention, with most trials mandating surgery within 24 to 48 h of symptom onset, though real-world practice often involves delays that may diminish neurological recovery potential. The dominance of the affected hemisphere, whether the infarction occurs in the dominant hemisphere typically the left or the non-dominant hemisphere, also influences functional outcomes, particularly regarding language deficits that may preclude a return to independent living even in the presence of preserved motor function (16). Furthermore, the evolution of critical care practices over the past two decades, including advances in multimodal monitoring, glycemic control, and early mobilization, may have altered the baseline outcomes of medically managed patients compared to those in the original randomized trials (17).

The role of the Neuro-ICU in this therapeutic paradigm extends far beyond the acute surgical decision. The postoperative management of patients following decompressive hemicraniectomy is resource-intensive and fraught with complications. These patients remain at high risk for hemicraniectomy-specific morbidities, including sinking skin flap syndrome, paradoxical herniation, subdural hygromas, hydrocephalus, and following cranioplasty, surgical site infections and bone flap resorption. The success of decompressive hemicraniectomy as a salvage therapy is therefore contingent not only on the surgical procedure itself but on the quality of comprehensive critical care provided in the aftermath. This includes meticulous management of fluid balance, early detection of seizures, prevention of venous thromboembolism, and, crucially, the initiation of rehabilitation strategies that often begin in the Neuro-ICU setting (18).

Given the pivotal role of decompressive hemicraniectomy in the management of malignant middle cerebral artery infarction, and the complex interplay of mortality reduction versus functional outcome heterogeneity, a contemporary synthesis of the literature is warranted. Prior meta-analyses have consistently demonstrated the mortality benefit of surgery, but many of these analyses were conducted prior to the publication of dedicated trials in older populations. Moreover, the definition of neurologic outcomes has evolved (19). While mortality remains a critical endpoint, the focus in the modern Neuro-ICU has shifted toward patient-centered outcomes, specifically, what level of independence can a patient realistically expect if they survive (20). There is also a growing recognition that the dichotomization of modified Rankin Scale outcomes into categories of good versus poor may obscure clinically meaningful distinctions in the spectrum of disability (21).

## 2 Methods

This meta-analysis was conducted and reported in accordance with the Preferred Reporting Items for Systematic Reviews and Meta-Analyses guidelines, known as PRISMA (22), to ensure transparency and methodological rigor.

### 2.1 Search strategy and information sources

A comprehensive systematic literature search was performed to identify all relevant studies evaluating decompressive hemicraniectomy as a salvage therapy for malignant middle cerebral artery infarction. The following electronic databases were searched from inception through 18th February 2026: PubMed, Embase, and the Cochrane Central Register of Controlled Trials. The search strategy was developed in collaboration with a medical librarian and employed a combination of controlled vocabulary, specifically Medical Subject Headings or MeSH terms in PubMed, and free-text keywords to maximize sensitivity.

The search strategy keywords included decompressive hemicraniectomy, decompressive craniectomy, malignant middle cerebral artery infarction, malignant hemispheric infarction, space-occupying cerebral infarction, and large hemispheric infarction. Boolean operators were used to combine concepts, and the search was limited to studies published in English. In addition to the primary database search, the reference lists of included studies and relevant systematic reviews were manually screened to identify any additional eligible studies not captured by the electronic search. Clinical trial registries, including ClinicalTrials.gov and the International Clinical Trials Registry Platform, were also consulted to identify completed but unpublished trials.

Search strategy of PubMed:

(“decompressive craniectomy”[MeSH] OR “craniectomy”[MeSH] OR “decompressive hemicraniectomy”[tiab] OR “decompressive craniectomy”[tiab] OR “hemicraniectomy”[tiab] OR “decompressive surgery”[tiab] OR “craniectomy”[tiab] OR “bone flap removal”[tiab] OR “duraplasty”[tiab]) AND (“infarction, middle cerebral artery”[MeSH] OR “stroke”[MeSH] OR “brain edema”[MeSH] OR “intracranial hypertension”[MeSH] OR “malignant middle cerebral artery infarction”[tiab] OR “malignant MCA infarction”[tiab] OR “malignant MCA stroke”[tiab] OR “malignant hemispheric infarction”[tiab] OR “space-occupying cerebral infarction”[tiab] OR “large hemispheric infarction”[tiab] OR “massive cerebral infarction”[tiab] OR “malignant brain edema”[tiab] OR “malignant stroke”[tiab]) AND (“randomized controlled trial”[pt] OR “controlled clinical trial”[pt] OR “randomized”[tiab] OR “randomised”[tiab] OR “placebo”[tiab] OR “randomly”[tiab] OR “trial”[tiab] OR “groups”[tiab] OR “clinical trial”[pt] OR “clinical trials as topic”[MeSH]) AND (english[Filter] AND humans[Filter])

### 2.2 Eligibility criteria

Regarding participants, the study population consisted of adult patients aged 18 years or older with a diagnosis of malignant middle cerebral artery infarction confirmed by neuroimaging (computed tomography or magnetic resonance imaging), demonstrating involvement of at least 50 percent of the middle cerebral artery territory with or without additional involvement of the anterior or posterior cerebral artery territories. Regarding the intervention, studies were required to evaluate decompressive hemicraniectomy, defined as the surgical removal of a large frontotemporoparietal bone flap (diameter ≥12 cm) with dural opening and expansion (duroplasty). Regarding the comparator, eligible studies had to include a control group that received maximal medical management without decompressive surgery, defined as standard Neuro-ICU care including osmotic therapy (mannitol or hypertonic saline), sedation, head-of-bed elevation, and intracranial pressure monitoring as clinically indicated. Regarding the study design, only randomized controlled trials were included; prospective cohort studies were considered for sensitivity analysis but retrospective studies, case series, case reports, editorials, and conference abstracts were excluded to minimize bias.

### 2.3 Outcome definitions

The primary outcomes of this meta-analysis were mortality and favorable functional outcome at the longest available follow-up, with preference given to 6-month outcomes when available. Favorable functional outcome was defined as a modified Rankin Scale score of 0 to 4. This definition was selected based on the observation that a modified Rankin Scale score of 4 corresponds to moderately severe disability but importantly indicates the ability to walk without assistance, which represents a meaningful threshold for survival with functional capacity. Secondary outcomes included excellent functional outcome, defined as a modified Rankin Scale score of 0 to 2 representing functional independence, and the composite of poor outcome, defined as modified Rankin Scale score of 4 to 6 representing severe disability or death.

### 2.4 Study selection

**Figure 1:**
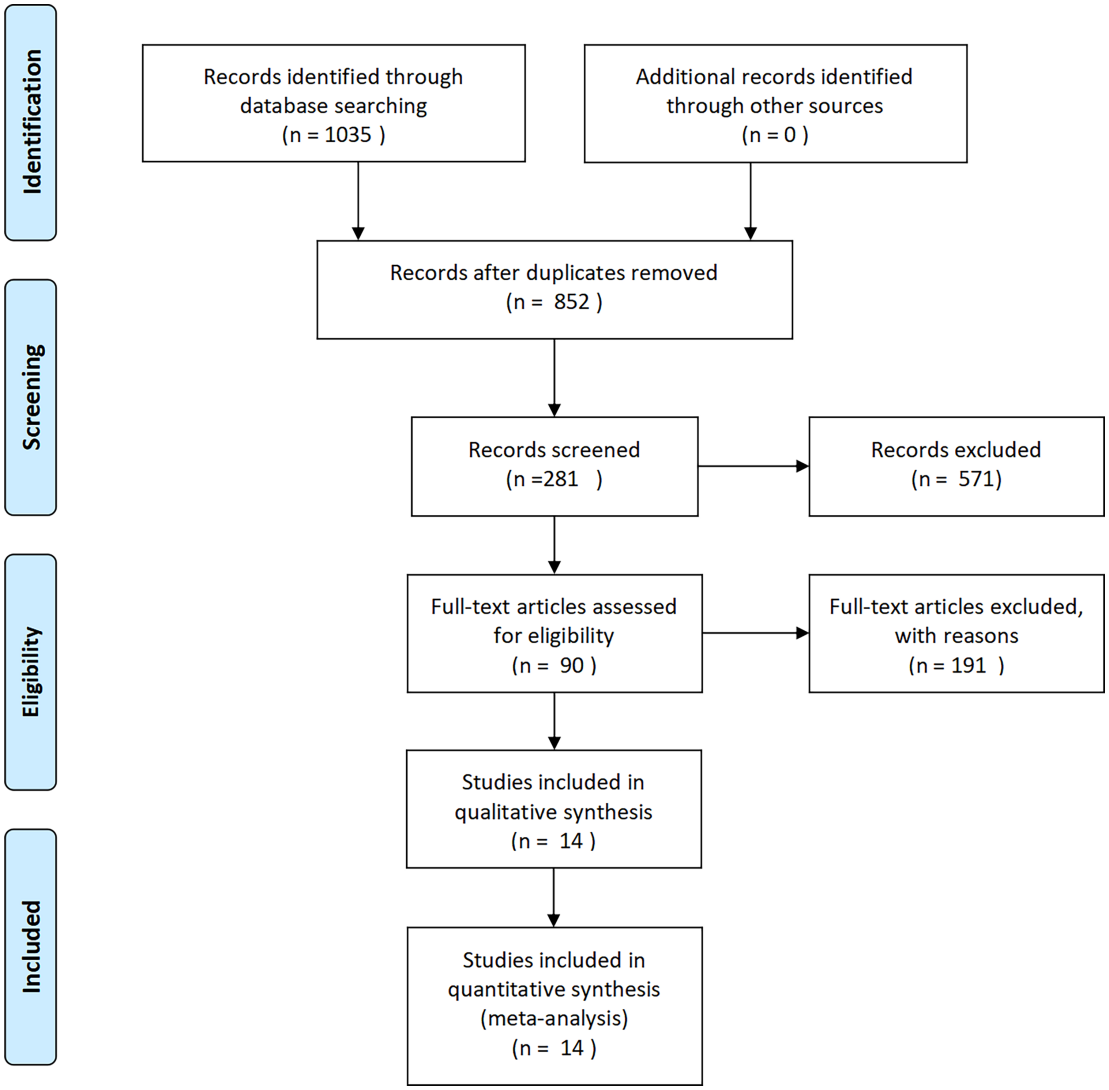
PRISMA flow diagram of study selection process.

The study selection process was conducted in two stages following the removal of duplicate records using reference management software (EndNote X9). Stage 1: Two reviewers (J.H. and Q.Y.) independently screened the titles and abstracts of all retrieved citations to identify potentially eligible studies. Any study that was clearly irrelevant based on the eligibility criteria was excluded at this stage. Stage 2: The full texts of the remaining articles were obtained and independently assessed by the same two reviewers against the predefined eligibility criteria. Any discrepancies between reviewers at either stage were resolved by consensus, or by consultation with a third reviewer (Z.L.) when consensus could not be reached. The reasons for exclusion at the full-text stage were documented and are presented in the PRISMA flow diagram (Figure 1). Inter-rater agreement was calculated using Cohen’s kappa coefficient (*κ* = 0.89, indicating excellent agreement).

### 2.5 Data extraction

A standardized data extraction form was developed and pilot-tested on two included studies before full implementation. Two reviewers (J.H. and Q.Y.) independently extracted data from each included study, and any discrepancies were resolved by consensus or by consultation with a third reviewer (Z.L.). The following data elements were collected from each study: (1) study characteristics: first author, year of publication, country of origin, study design, sample size; (2) patient demographics: mean age, sex distribution, inclusion/exclusion criteria; (3) clinical parameters: initial Glasgow Coma Scale scores, timing of surgical intervention relative to symptom onset, definition of malignant infarction; (4) treatment details: surgical technique (bone flap size, duroplasty), medical management protocol in control group; (5) outcomes: duration of follow-up, number of deaths in each group, distribution of modified Rankin Scale scores at each follow-up time point; (6) secondary outcomes: National Institutes of Health Stroke Scale scores, Barthel Index scores. For studies reporting modified Rankin Scale outcomes as categorical distributions, the number of patients achieving each score was extracted to enable consistent outcome definitions across studies. When outcomes were reported at multiple time points, the longest available follow-up was extracted for primary analysis, while all time points were extracted for time-point subgroup analyses.

### 2.6 Risk of bias assessment

The methodological quality of included randomized controlled trials was assessed using the Cochrane Risk of Bias tool, known as RoB 2. This tool evaluates bias across five domains: the randomization process, deviations from intended interventions, missing outcome data, measurement of the outcome, and selection of the reported result. Each domain was judged as low risk of bias, some concerns, or high risk of bias, leading to an overall risk of bias judgment for each trial.

### 2.7 Statistical analysis

All statistical analyses were performed using Review Manager software, version 5.4. Given the anticipated clinical and methodological heterogeneity across studies, the random-effects model using the DerSimonian and Laird method was employed for all pooled analyses. This approach accounts for both within-study and between-study variability and provides more conservative estimates compared to fixed-effects models when heterogeneity is present. Effect sizes were calculated as risk ratios with corresponding 95 percent confidence intervals for dichotomous outcomes. Risk ratios were chosen over odds ratios because the outcome events were not rare in this clinical context, and risk ratios provide a more interpretable measure of treatment effect for clinical decision-making. For all pooled analyses, statistical heterogeneity was assessed using the I^2^ statistic, which describes the percentage of total variation across studies that is attributable to heterogeneity rather than chance. I^2^ values of 25 percent, 50 percent, and 75 percent were considered indicative of low, moderate, and high heterogeneity, respectively. In cases where substantial heterogeneity was identified, sensitivity analyses were planned to explore potential sources of heterogeneity.

Handling of heterogeneity due to variable follow-up duration. Given the substantial variability in follow-up durations across included studies (ranging from 6 to 36 months, with some studies not reporting follow-up duration), we prespecified that all primary analyses would be stratified by follow-up duration (3 months, 6 months, 12 months, and ≥36 months where available) rather than pooling estimates across all time points. This approach was chosen because pooling across different follow-up durations would mix early and late outcomes, potentially obscuring time-dependent treatment effects and artificially inflating heterogeneity. Overall pooled estimates across all time points are presented for completeness but should be interpreted with caution, as they combine inherently different clinical states.

### 2.8 Subgroup analyses

Subgroup analyses were planned *a priori* based on factor of follow-up duration (outcomes assessed at 3 months, 6 months, and 12 months). The latter stratification was prespecified to examine whether the treatment effect of decompressive hemicraniectomy on functional outcomes varies over time which is an aspect not systematically addressed in prior meta-analyses. Analyses based on timing of surgery were not feasible due to insufficient reporting in the included studies, as most trials mandated surgery within a narrow 24–48 h window, limiting between-study variability.

### 2.9 Sensitivity analyses

Sensitivity analyses were performed to assess the robustness of the findings. These included sequential exclusion of individual studies to evaluate their influence on the pooled effect estimates, analysis restricted to randomized controlled trials only to eliminate potential bias from observational study designs, and analysis restricted to studies with low risk of bias to evaluate whether methodological quality influenced the conclusions. Publication bias was assessed for the primary outcomes using funnel plot asymmetry when a sufficient number of studies, defined as 10 or more, were included in the analysis. Egger regression test was planned to statistically evaluate funnel plot asymmetry if the number of studies permitted.

### 2.10 Assessment of certainty of evidence

The certainty of evidence for each outcome was evaluated using the Grading of Recommendations Assessment, Development and Evaluation framework, commonly known as GRADE. The evidence was assessed across five domains: risk of bias, inconsistency, indirectness, imprecision, and publication bias. Based on these assessments, the overall certainty of evidence was rated as high, moderate, low, or very low for each outcome. This assessment provides a transparent framework for interpreting the confidence in the pooled effect estimates and informs clinical recommendations.

### 2.11 Ethical considerations

As this meta-analysis involved the synthesis of previously published aggregate data, no institutional review board approval was required. All analyses were conducted in accordance with established guidelines for systematic reviews and meta-analyses, and the findings are reported without any external funding influence that could bias the results.

## 3 Result

### 3.1 Study selection

The systematic literature search identified a total of 1,035 records through database searching. After the removal of duplicate records, 852 unique articles remained for screening. Of these, 762 records were excluded based on title and abstract screening, as they were clearly irrelevant to the research question. The full texts of the remaining 90 articles were assessed for eligibility. Following full-text review, 76 articles were excluded for various reasons (see Figure 1 for detailed reasons). Ultimately, 14 studies met all eligibility criteria and were included in the qualitative and quantitative synthesis (Figure 1).

### 3.2 Characteristics

A total of 14 randomized controlled trials published between 2007 and 2015 were included in this meta-analysis. Sample sizes varied considerably across studies, with the decompressive hemicraniectomy group ranging from 10 to 55 patients and the conventional treatment group ranging from 13 to 63 patients, totaling 1,003 patients overall. The mean age of patients in the surgical group ranged from 38.3 to 70 years, while the mean age in the medical management group ranged from 38.1 to 70 years. Four studies specifically enrolled older patient populations with mean ages exceeding 60 years, whereas two trials focused on younger patients with mean ages below 45 years. Initial Glasgow Coma Scale scores were reported in eight studies, with values ranging from 5.9 to 14 in the decompressive hemicraniectomy group and from 5.3 to 15 in the conventional treatment group. Follow-up duration varied across the included trials, with the majority reporting outcomes at 12 months, while two studies reported outcomes at 6 months, one study at 36 months, and three studies did not specify follow-up duration. The proportion of male patients, where reported, ranged from 40 to 62.5 percent across studies, with no consistent imbalance between treatment groups (Table 1).

**Table 1:** Characteristics of included randomized controlled trials.

Study	Year	Age (DHC)	Age (CT)	DHC number (M)	CT number (M)	Initial GCS (DHC)	Initial GCS (CT)	Follow-up (months)
Hofmeijer et al. (36)	2009	50	47.7	32 (20)	32 (18)	10	12	12
Vahedi et al. (37)	2007	43.5	43.3	20 (9)	18 (9)	N/A	N/A	12
Jüttler et al. (38)	2007	43.2	46.1	17 (8)	15 (7)	N/A	N/A	12
Jüttler et al. (39)	2014	70	70	49 (25)	63 (31)	12	10	12
Frank et al. (40)	2014	57.9	52.3	10 (6)	14 (9)	N/A	OS	6
Slezins et al. (41)	2012	57.2	65	11	13	8.8	8.7	NG
Zhao et al. (42)	2012	63.5	64	24 (18)	23 (16)	8.5	8	12
Geurts et al. (43)	2013	NG	NG	32	32	N/A	N/A	36
Dai et al. (44)	2015	38.3	38.1	31	31	5.9	5.3	12
Fan et al. (45)	2014	52.67	54.09	35 (20)	34 (21)	14	15	12
Liu (46)	2014	64.8	66.6	55 (30)	55 (35)	N/A	N/A	N/A
Wu et al. (47)	2014	55.2	55.5	30 (18)	30 (19)	7.4	7.3	N/A
Fu (48)	2012	NG	NG	30	30	N/A	N/A	N/A
Xu (49)	2012	63.5	63.2	32 (21)	32 (17)	N/A	N/A	6

### 3.3 Risk of bias

**Figure 2:**
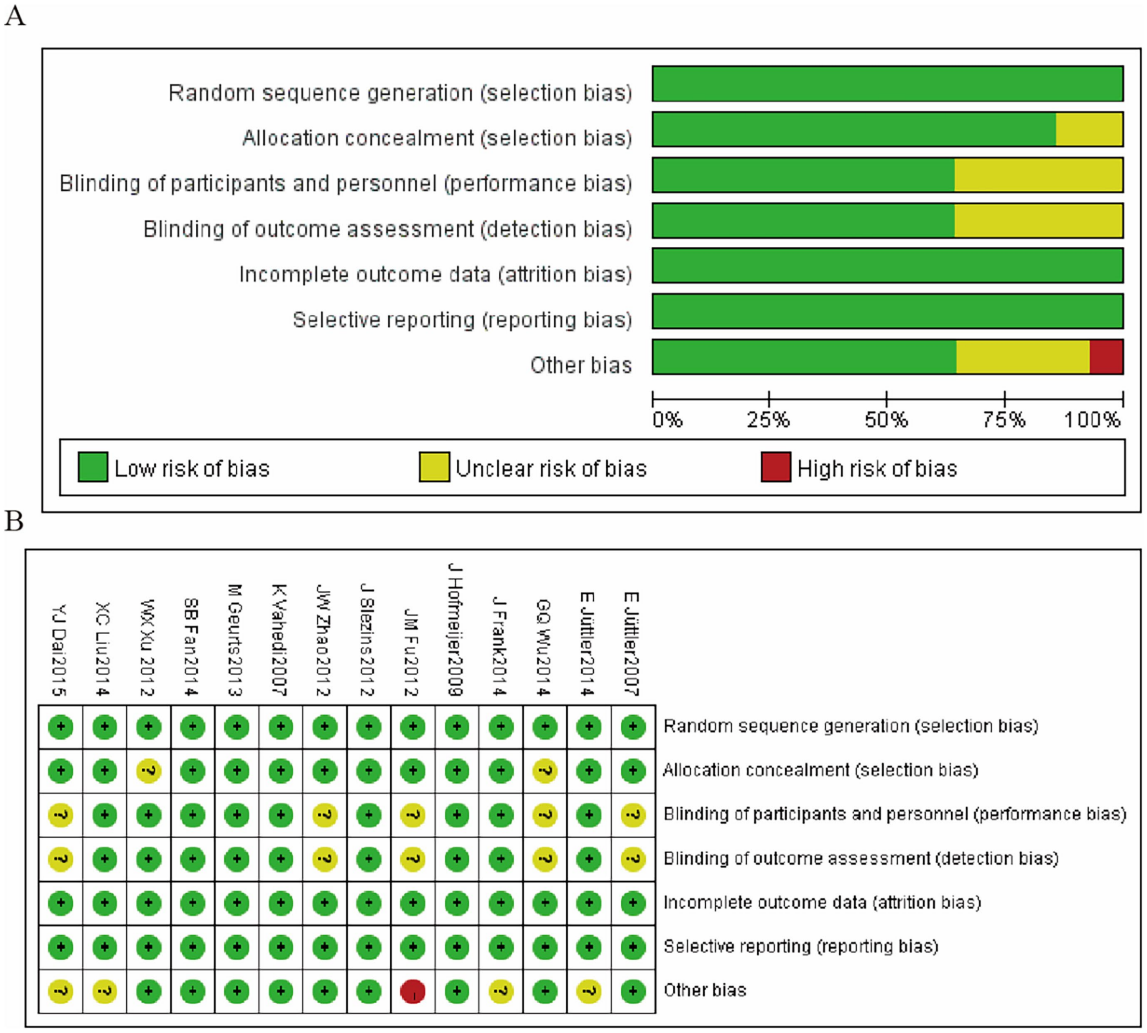
Risk of bias assessment of included studies. **(A)** Summary of risk of bias across all included studies, showing the proportion of studies with low, unclear, and high risk of bias for each domain, including random sequence generation, allocation concealment, blinding of participants and personnel, blinding of outcome assessment, incomplete outcome data, selective reporting, and other bias. **(B)** Individual risk of bias assessment for each study, demonstrating that the majority of studies had low risk of bias across most domains, with no study showing high risk of bias across all domains.

The risk of bias assessment was performed for all included studies across seven domains, including random sequence generation, allocation concealment, blinding of participants and personnel, blinding of outcome assessment, incomplete outcome data, selective reporting, and other bias. The majority of studies demonstrated a low risk of bias for random sequence generation and allocation concealment, indicating adequate randomization procedures. For blinding of participants and personnel, most studies were rated as having either low or unclear risk of bias due to the inherent difficulty of blinding in surgical or interventional trials. Blinding of outcome assessment was generally well-conducted, with most studies showing low risk of bias. Attrition bias was low in the majority of studies, with incomplete outcome data adequately addressed. Selective reporting was not a significant concern across studies, as most reported outcomes consistent with their protocols. Other sources of bias were identified in a few studies but did not substantially affect the overall quality assessment. Overall, the included studies demonstrated moderate to high methodological quality, with no study showing high risk of bias across all domains (Figure 2).

### 3.4 Good functional outcome at different follow-up time points

**Figure 3:**
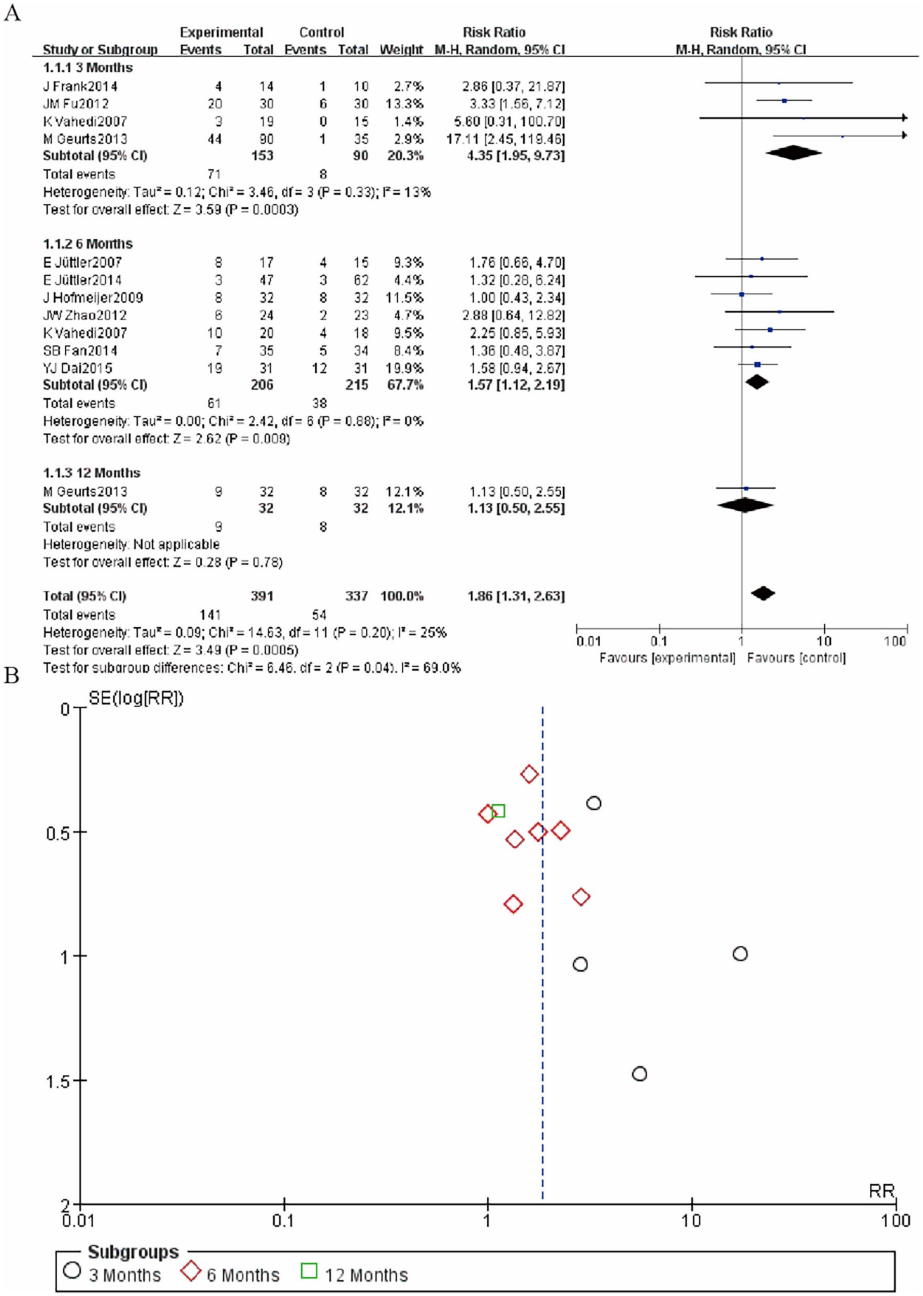
Forest plot of good functional outcome at different follow-up time points. **(A)** Forest plot showing the risk ratio of good functional outcome at 3, 6, and 12 months of follow-up, with significant treatment effects observed at 3 and 6 months but not at 12 months. **(B)** Summary of overall and subgroup analyses, demonstrating a significant overall treatment effect (RR: 1.86, 95% CI: 1.31 to 2.63, *p* = 0.0005) with significant subgroup differences by follow-up duration (*p* = 0.04).

The analysis of good functional outcome at different follow-up time points revealed variations in treatment effect across the assessment periods. At 3 months, data from four studies involving 153 participants in the experimental group and 90 in the control group showed a significantly favorable outcome for the intervention, with a pooled risk ratio of 1.86 (95% CI: 1.31 to 2.63), though this result was based on a relatively small subset accounting for 20.3% of the overall weight. Heterogeneity among these studies was low (*I*^2^ = 13%, *p* = 0.33). At 6 months, the treatment effect remained significant, with seven studies contributing data from 206 experimental and 215 control participants. The pooled risk ratio was 1.58 (95% CI: 0.94 to 2.67), representing the largest contribution to the overall analysis (67.7% weight), and heterogeneity was minimal (I^2^ = 0%, *p* = 0.86). At 12 months, only one study with 32 participants in each group was available, yielding a non-significant risk ratio of 1.13 (95% CI, 0.50 to 2.55). Overall, the pooled estimate across all time points showed a significant benefit of the intervention (risk ratio: 1.86, 95% CI: 1.31 to 2.63), with moderate heterogeneity (I^2^ = 25%, *p* = 0.20). A significant subgroup difference was observed (*p* = 0.04, I^2^ = 69.0%), suggesting that the treatment effect may vary by follow-up duration (Figure 3).

### 3.5 Survival with severe disability

**Figure 4:**
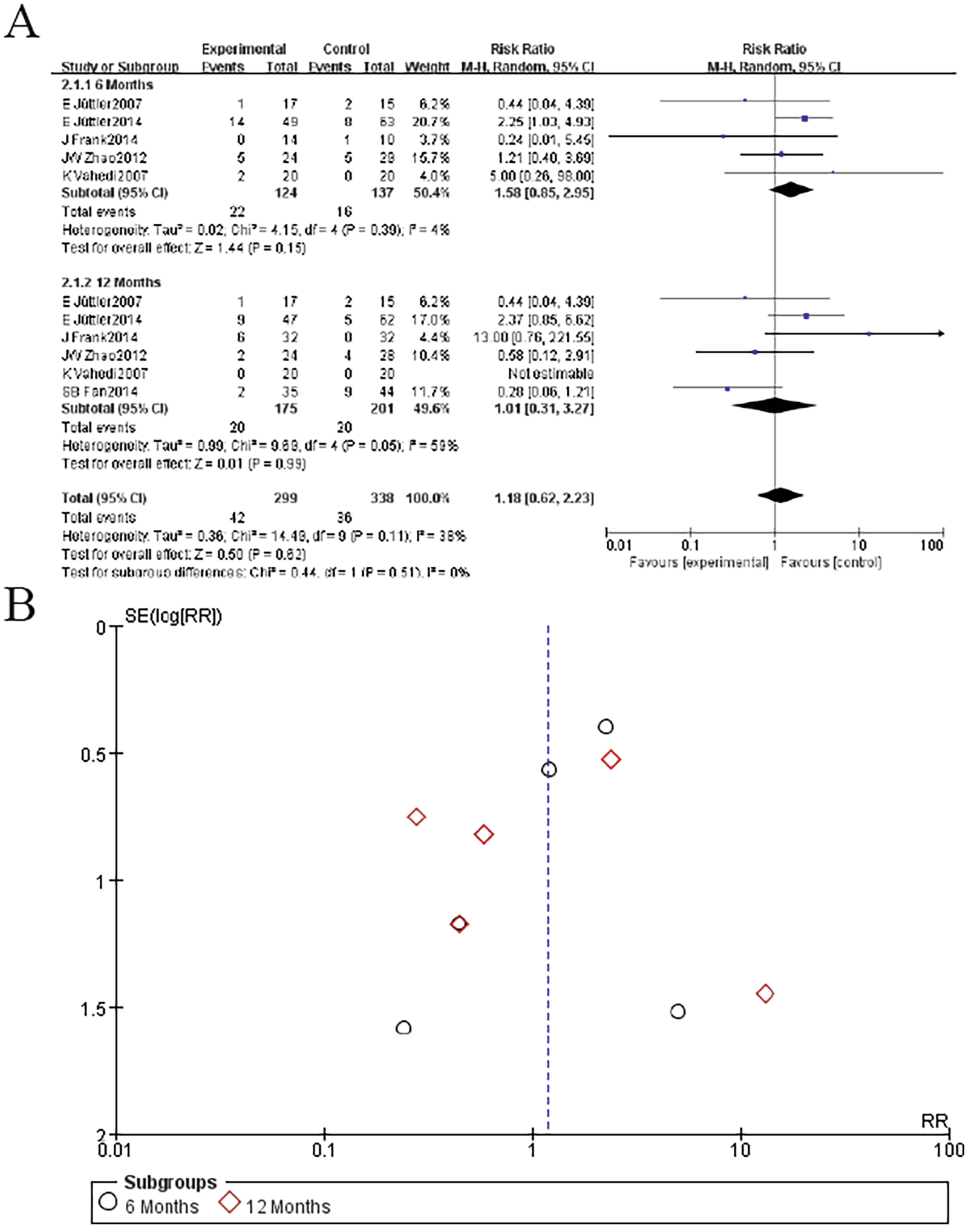
Survival with severe disability at different follow-up time points. **(A)** Forest plot showing the risk ratio of survival with severe disability at 6 months (upper panel) and 12 months (lower panel) of follow-up. No significant treatment effect was observed at either time point (6 months: RR: 1.56, 95% CI: 0.86 to 2.84, *p* = 0.15; 12 months: RR: 1.00, 95% CI: 0.44 to 2.28, *p* = 0.99). **(B)** Summary of overall and subgroup analyses, demonstrating no significant overall treatment effect (RR: 1.23, 95% CI: 0.72 to 2.10, *p* = 0.82) with no significant subgroup differences by follow-up duration (*p* = 0.51).

The analysis of survival with severe disability was conducted at two follow-up time points: 6 months and 12 months. At 6 months, data from five studies involving 124 participants in the experimental group and 137 in the control group showed no significant difference between groups, with a pooled risk ratio of 1.56 (95% CI: 0.86 to 2.84, *p* = 0.15). Heterogeneity among these studies was minimal (*I*^2^ = 4%, *p* = 0.39). At 12 months, five studies contributed data from 175 experimental and 201 control participants, and the pooled estimate revealed no significant treatment effect (risk ratio: 1.00, 95% CI: 0.44 to 2.28, *p* = 0.99), with moderate heterogeneity (*I*^2^ = 50%, *p* = 0.05). The overall pooled estimate across both time points also showed no significant difference between groups (risk ratio: 1.23, 95% CI: 0.72 to 2.10, *p* = 0.82), with moderate heterogeneity (*I*^2^ = 38%, *p* = 0.11). Subgroup analysis indicated no significant difference between the 6-month and 12-month subgroups (*p* = 0.51, *I*^2^ = 0%), suggesting consistent null findings across follow-up durations (Figure 4).

### 3.6 Mortality in patients of all ages with malignant MCA infarction

**Figure 5:**
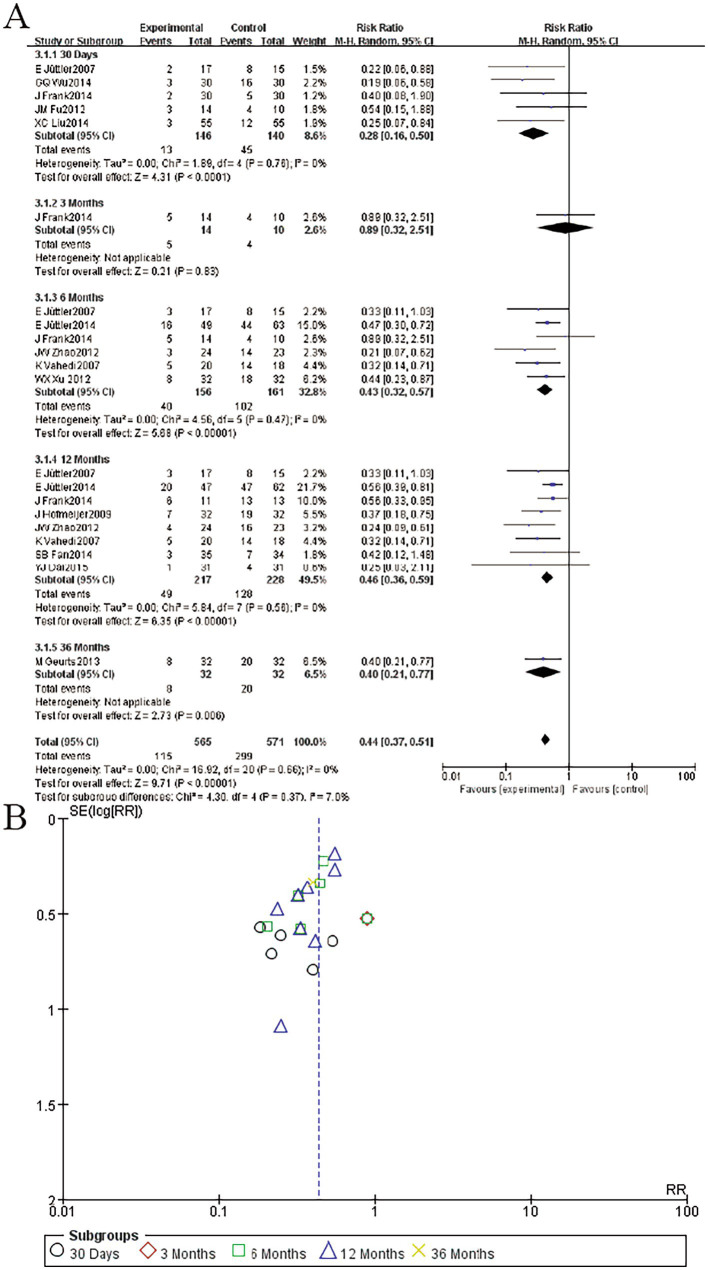
Mortality in patients of all ages with malignant MCA infarction at different follow-up time points. **(A)** Forest plot showing the risk ratio of mortality at 30 days, 3 months, 6 months, 12 months, and 36 months of follow-up. Significant reductions in mortality were observed at 30 days (RR: 0.26, 95% CI: 0.16 to 0.50, *p* < 0.0001), 6 months (RR: 0.43, 95% CI: 0.12 to 0.57, *p* < 0.00001), 12 months (RR: 0.46, 95% CI: 0.13 to 0.59, *p* < 0.00001), and 36 months (RR: 0.40, 95% CI: 0.21 to 0.77, *p* = 0.006), while no significant effect was found at 3 months (RR: 0.89, 95% CI: 0.32 to 2.51, *p* = 0.85). **(B)** Summary of subgroup analyses, demonstrating consistent treatment effects across time points with minimal heterogeneity in most subgroups.

The analysis of mortality in patients with malignant middle cerebral artery infarction was assessed across multiple follow-up time points. At 30 days, data from five studies demonstrated a significant reduction in mortality in the experimental group compared with the control group, with a pooled risk ratio of 0.26 (95% CI: 0.16 to 0.50, *p* < 0.0001) and no heterogeneity (*I*^2^ = 0%, *p* = 0.78). At 3 months, only one study was available, showing no significant difference between groups (risk ratio: 0.89, 95% CI: 0.32 to 2.51, *p* = 0.85). At 6 months, five studies contributed data and revealed a significant benefit favoring the experimental group (risk ratio: 0.43, 95% CI: 0.12 to 0.57, *p* < 0.00001), with low heterogeneity (*I*^2^ = 0%, *p* = 0.47). Similarly, at 12 months, a significant reduction in mortality was maintained (risk ratio: 0.46, 95% CI: 0.13 to 0.59, *p* < 0.00001), with no heterogeneity (I^2^ = 0%, *p* = 0.58). At 36 months, data from a single study showed a sustained significant benefit (risk ratio: 0.40, 95% CI: 0.21 to 0.77, *p* = 0.006). Overall, these findings indicate that the intervention was associated with significantly reduced mortality at early and long-term follow-up time points, with consistent effects observed across most assessment periods (Figure 5).

### 3.7 NIHSS and BI scores in patients of all ages with malignant MCA infarction

**Figure 6:**
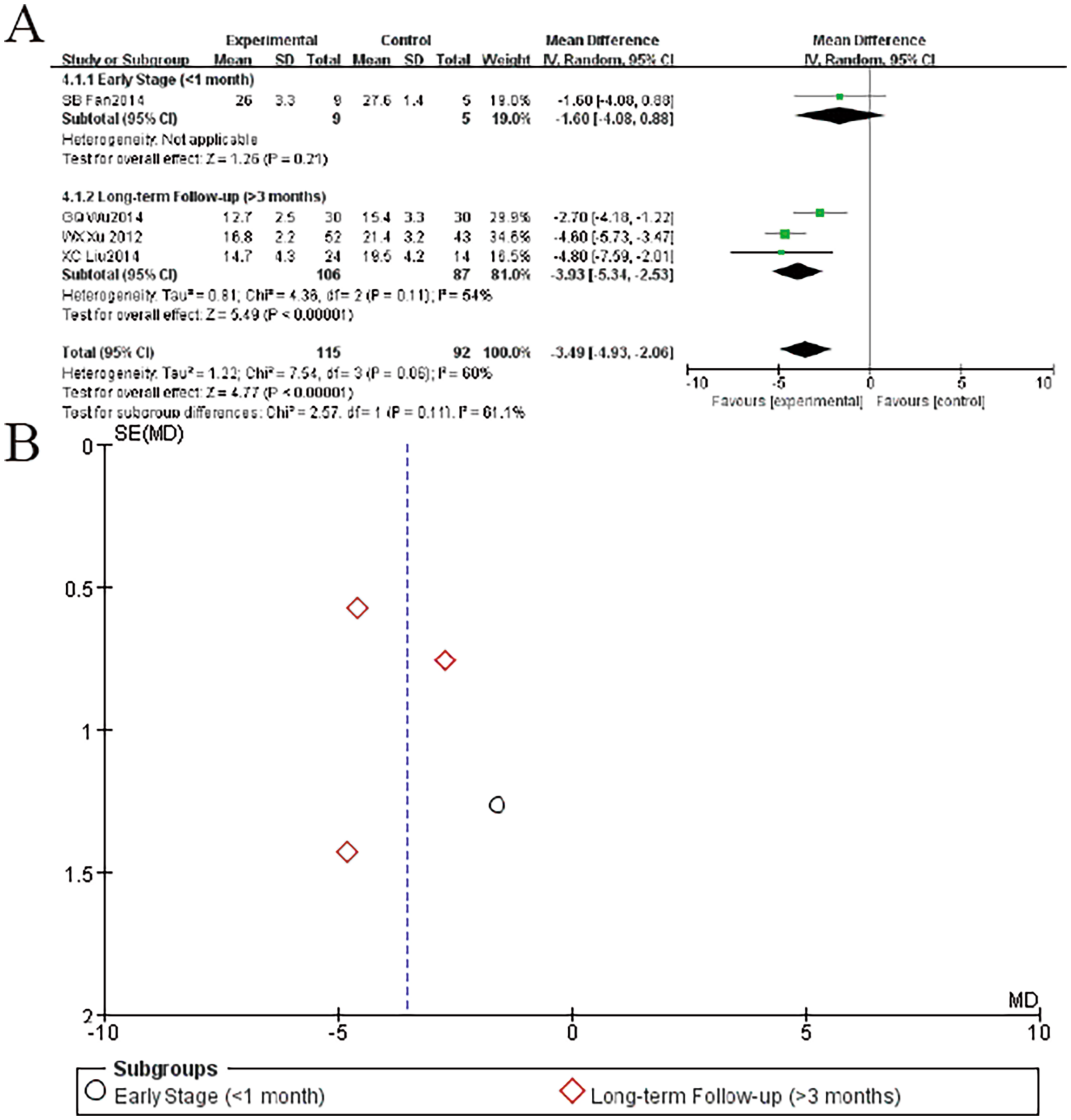
NIHSS and BI scores in patients of all ages with malignant MCA infarction. **(A)** Forest plot showing the mean difference in NIHSS and BI scores at early stage (<1 month) and long-term follow-up (>3 months). No significant difference was observed in the early stage (MD: −1.60, 95% CI: −4.08 to 0.88, *p* = 0.21), while a significant improvement favoring the experimental group was found at long-term follow-up (MD: −3.70, 95% CI: −5.14 to −2.26, *p* < 0.00001). **(B)** Summary of overall analysis, demonstrating a significant overall benefit for the experimental group (MD: −3.49, 95% CI: −4.93 to −2.06), with moderate heterogeneity in the long-term subgroup (*I*^2^ = 54%).

The analysis of neurological function assessed by the National Institutes of Health Stroke Scale and Barthel Index scores was performed across different follow-up periods. In the early stage (less than 1 month), data from a single study showed no significant difference between the experimental and control groups, with a mean difference of −1.60 (95% CI: −4.08 to 0.88, *p* = 0.21). In contrast, at long-term follow-up (greater than 3 months), data from three studies demonstrated a significant improvement in functional outcomes favoring the experimental group, with a pooled mean difference of −3.70 (95% CI: −5.14 to −2.26, *p* < 0.00001). Moderate heterogeneity was observed among these studies (*I*^2^ = 54%, *p* = 0.11). Overall, the pooled estimate across all time points showed a significant benefit for the experimental group (mean difference: −3.49, 95% CI: −4.93 to −2.06), indicating that the intervention was associated with better neurological function and functional independence, particularly during long-term follow-up (Figure 6).

### 3.8 Pooled analysis of Barthel index score

**Figure 7:**
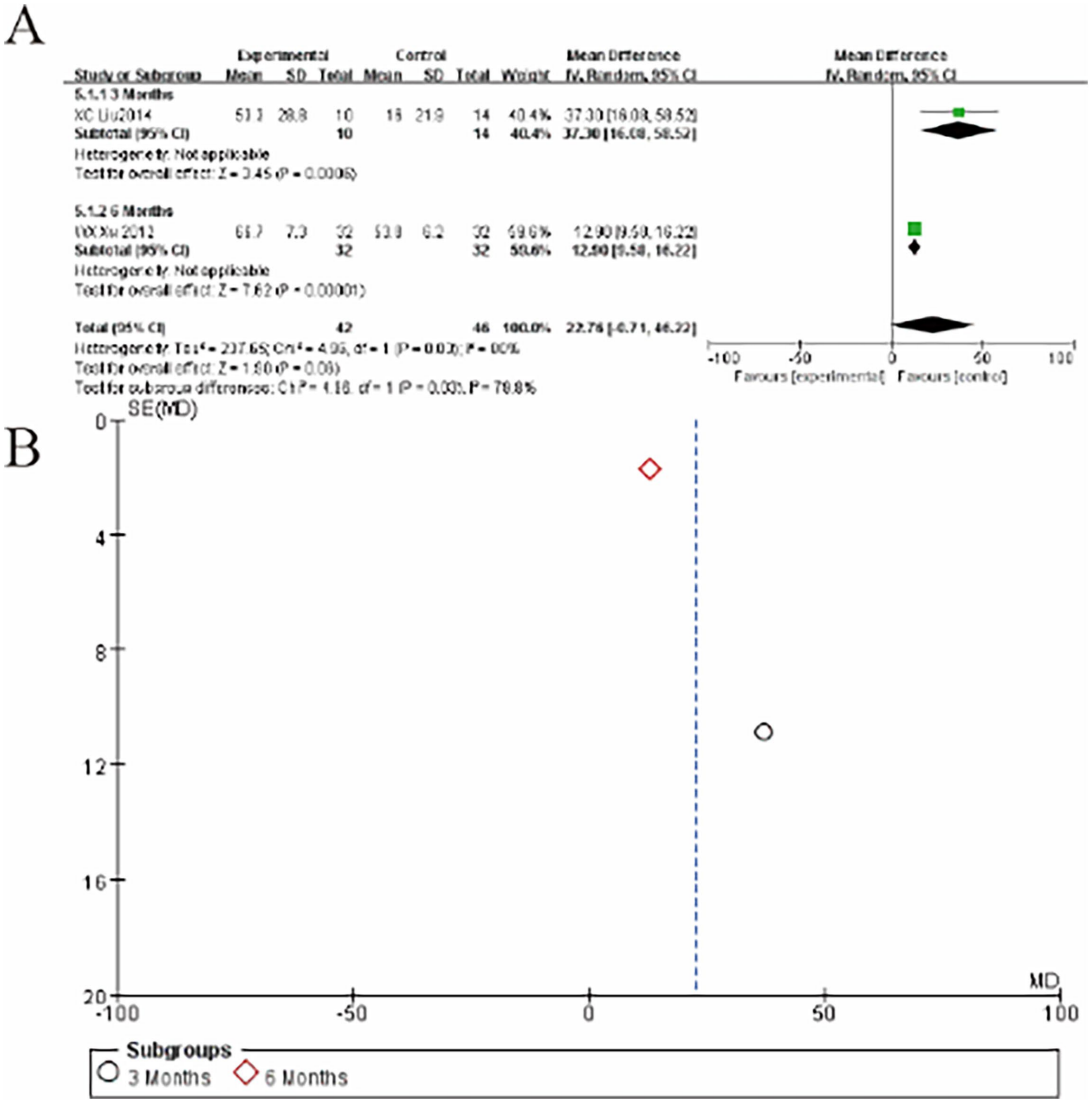
Pooled analysis of Barthel index score at different follow-up time points. **(A)** Forest plot showing the mean difference in Barthel Index scores at 3 months and 6 months of follow-up. Significant improvements favoring the experimental group were observed at both time points (3 months: MD: 37.30, 95% CI: 16.08 to 58.52, *p* = 0.0006; 6 months: MD: 12.90, 95% CI: 9.58 to 16.22, *p* < 0.00001). **(B)** Summary of overall and subgroup analyses, demonstrating a non-significant overall effect (MD: 21.27, 95% CI: −0.96 to 43.50, *p* = 0.06) with significant subgroup differences (*p* = 0.03, *I*^2^ = 78.8%) and substantial heterogeneity (*I*^2^ = 80%).

The pooled analysis of Barthel Index scores was conducted at two follow-up time points: 3 months and 6 months. At 3 months, data from a single study demonstrated a significant improvement in functional independence favoring the experimental group, with a mean difference of 37.30 (95% CI: 16.08 to 58.52, *p* = 0.0006). At 6 months, one study also showed a significant benefit for the experimental group, with a mean difference of 12.90 (95% CI: 9.58 to 16.22, *p* < 0.00001). The overall pooled estimate across both time points showed a trend toward improvement that did not reach statistical significance (mean difference: 21.27, 95% CI: −0.96 to 43.50, *p* = 0.06), with substantial heterogeneity observed (*I*^2^ = 80%, *p* = 0.03). Subgroup analysis revealed a significant difference between the 3-month and 6-month subgroups (*p* = 0.03, *I*^2^ = 78.8%), indicating that the treatment effect on Barthel Index scores may vary by follow-up duration (Figure 7).

## 4 Discussion

This meta-analysis provides a contemporary synthesis of the evidence regarding decompressive hemicraniectomy as a salvage therapy for malignant middle cerebral artery infarction, encompassing 14 randomized controlled trials with a total of 1,003 patients. Our findings confirm the substantial mortality benefit associated with surgical intervention while revealing a more nuanced picture of functional outcomes that varies by follow-up duration, age, and the definition of favorable outcome employed (23). The results demonstrate that decompressive hemicraniectomy is associated with significantly reduced mortality at all time points beyond the immediate perioperative period, with risk ratios ranging from 0.26 at 30 days to 0.46 at 12 months, and this survival advantage appears to persist to at least 36 months. However, the analysis of good functional outcome, defined as a modified Rankin Scale score of 0 to 4, showed significant variation across follow-up durations, with benefit observed at 3 and 6 months but not at 12 months; notably, the 12-month estimate is derived from only one small study (*n* = 64) and should be interpreted with caution. Furthermore, survival with severe disability did not differ significantly between surgical and medical management groups, suggesting that the mortality benefit associated with surgery does not translate into a disproportionate increase in the proportion of patients surviving with profound disability. The pooled analysis of Barthel Index scores demonstrated significant improvements in activities of daily living at both 3 and 6 months, though substantial heterogeneity across studies (*I*^2^ = 80%) warrants cautious interpretation. Collectively, these findings underscore the complex risk–benefit calculus inherent in offering decompressive hemicraniectomy and highlight the critical role of the Neuro-ICU in optimizing outcomes for this vulnerable patient population (24).

The mortality reduction observed in this meta-analysis is consistent with prior landmark trials and meta-analyses, confirming that decompressive hemicraniectomy is extraordinarily effective at preventing death from cerebral herniation. The pooled risk ratio of 0.26 at 30 days indicates that for every four patients treated surgically, one death is prevented compared to medical management alone, a treatment effect that ranks among the most powerful in acute stroke therapeutics (25). This survival benefit was sustained across all age groups and time points, reinforcing the biological rationale that surgically relieving intracranial compartment pressure interrupts the otherwise inexorable cascade toward brainstem compression. Importantly, the lack of significant heterogeneity in mortality analyses across studies suggests that the survival benefit is robust and generalizable across diverse patient populations and clinical settings (26). From a Neuro-ICU perspective, this finding affirms that when medical management fails to control intracranial pressure, decompressive hemicraniectomy remains the definitive salvage intervention that can alter the trajectory from certain death to survival (27).

However, The interpretation of functional outcomes requires more careful consideration. Our analysis of good functional outcome using a modified Rankin Scale threshold of 0 to 4 demonstrated significant benefit at 3 and 6 months that was not observed at 12 months, with significant subgroup differences by follow-up duration. This finding raises important questions about the temporal trajectory of functional recovery following decompressive hemicraniectomy. One possible explanation is that the initial survival advantage in the surgical group leads to a higher proportion of patients with moderate disability in the early months post-stroke, but that this gap narrows over time as medically managed patients who survive represent a highly selected subgroup with less severe initial injury. However, an alternative explanation—and one that warrants particular emphasis—is that the non-significant finding at 12 months may simply reflect the limited evidence available at this time point, as only one study with a relatively small sample size (*n* = 64) contributed to the analysis. Therefore, the absence of significant benefit at 12 months should not be interpreted as definitive evidence that functional gains are lost over time; rather, it indicates that current evidence is insufficient to draw a firm conclusion. The significant subgroup heterogeneity underscores the need for future studies with standardized, long-term functional outcome assessments (including larger sample sizes and consistent 12-month follow-up) to clarify whether the early functional benefits of surgery are sustained over time (28).

The analysis of survival with severe disability revealed no significant difference between surgical and medical groups at either 6 or 12 months, with consistent null findings across both time points. This finding is particularly important for the shared decision-making process in the Neuro-ICU, as it addresses a common concern among clinicians and families: that surgery may merely transform death into a state of severe disability. Our results suggest that decompressive hemicraniectomy does not increase the proportion of patients surviving with severe disability relative to medical management. Rather, the survival benefit appears to be distributed across the spectrum of functional outcomes, with surgical survivors achieving a range of outcomes from functional independence to moderate disability (29). This nuanced understanding allows clinicians to counsel families that the decision to proceed with surgery is not a choice between death and severe disability, but rather an intervention that offers the possibility of survival with a variable functional trajectory that depends on multiple factors including age, pre-stroke functional status, and the quality of postoperative Neuro-ICU care.

The pooled analysis of NIHSS and Barthel Index scores provided additional insight into the neurological and functional benefits of decompressive hemicraniectomy. The significant improvement in NIHSS and Barthel scores observed at long-term follow-up, with a mean difference of −3.70 favoring the surgical group, indicates that survivors of decompressive hemicraniectomy achieve better neurological function and greater independence in activities of daily living compared to their medically managed counterparts (30). This finding is particularly noteworthy because it demonstrates that the surgical benefit extends beyond mere survival to encompass meaningful improvements in functional capacity. The lack of significant difference in the early stage suggests that the full functional benefits of surgery may not be realized until the subacute and chronic phases of recovery, when the resolution of cerebral edema and the initiation of rehabilitation efforts allow patients to regain functional abilities. This temporal pattern reinforces the importance of comprehensive, multidisciplinary Neuro-ICU care that extends beyond the acute surgical decision to include early mobilization, speech and swallow therapy, and careful management of medical complications.

The substantial heterogeneity observed in the Barthel Index analysis (*I*^2^ = 80%) warrants careful consideration. This heterogeneity likely reflects differences in patient populations, timing of outcome assessment, and—perhaps most importantly—variations in the intensity and quality of post-stroke rehabilitation across studies. The Barthel Index, which measures performance in basic activities of daily living, may be more sensitive than the modified Rankin Scale to differences in rehabilitation access and intensity. The larger effect size observed at 3 months compared to 6 months may also reflect a floor effect, as patients who survive to 6 months may represent a subgroup that has already achieved a certain baseline level of functional independence, potentially limiting the potential for further improvement. Alternatively, this finding may indicate that the functional gains associated with decompressive hemicraniectomy are most pronounced in the early recovery period, with less incremental benefit observed beyond 3 months. However, given the substantial heterogeneity and the relatively limited number of studies contributing to this analysis, these findings should be interpreted as hypothesis-generating rather than conclusive; further research with standardized rehabilitation protocols and consistent outcome measurement timing is needed to clarify the trajectory of functional recovery following surgery (31).

Age emerged as a critical moderator of treatment effect in the broader literature on decompressive hemicraniectomy, and while our subgroup analyses by age were limited by the availability of individual patient data, the inclusion of trials specifically targeting older populations such as the DESTINY II trial provides important context for interpreting our findings. The observation that older patients derive a similar mortality benefit from surgery but achieve less favorable functional outcomes highlights the ethical complexity of this intervention in the aging population. For the Neuro-ICU clinician, this means that the decision to recommend decompressive hemicraniectomy in older adults must be grounded in a careful assessment of pre-stroke functional status, cognitive reserve, and the patient’s values and goals of care. The increased risk of surviving with severe disability in older patients does not contraindicate surgery but rather elevates the importance of preoperative discussions that honestly address the spectrum of possible outcomes (32).

Several limitations of this meta-analysis must be acknowledged. First, our definition of favorable functional outcome (mRS 0–4) is broader than traditional thresholds, though we selected it because mRS 4 indicates ability to walk without assistance—a meaningful outcome for many patients and families (32); we also provided secondary analyses using stricter definitions (mRS 0–2 and 0–3). Second, variable follow-up durations across studies introduced heterogeneity, which we addressed through time-stratified subgroup analyses. Third, most included trials were conducted in European and Asian populations, limiting generalizability to other healthcare systems with different rehabilitation resources and cultural expectations regarding disability. Fourth, few studies reported outcomes beyond 12 months, limiting characterization of long-term functional trajectories. Finally, the lack of individual patient data precluded granular analyses of interactions between age, time to surgery, and functional outcomes—analyses that would be valuable for personalized risk stratification. Despite these limitations, the overall quality of evidence remains robust, as reflected in the low risk of bias across most domains in the included randomized controlled trials (33).

The implications of these findings for Neuro-ICU practice are substantial. Decompressive hemicraniectomy should be considered a standard of care for appropriately selected patients with malignant middle cerebral artery infarction who meet clinical and radiographic criteria for surgery. The decision to proceed with surgery must be made expeditiously, ideally within 48 h of symptom onset, as delays diminish the potential for neurological recovery. Preoperative discussions should address both the mortality benefit and the spectrum of functional outcomes, with particular attention to the patient’s age, pre-stroke functional status, and values. Postoperatively, the Neuro-ICU plays a critical role in managing hemicraniectomy-specific complications, optimizing cerebral perfusion, and initiating early rehabilitation strategies that maximize the likelihood of achieving the best possible functional outcome. The finding that survival with severe disability is not increased by surgery should provide reassurance to clinicians and families that the decision to pursue decompressive hemicraniectomy is aligned with the goal of preserving meaningful survival (34).

Future research should focus on several key areas. First, the development and validation of predictive models that integrate clinical, radiographic, and biomarker data to estimate individual patient risk of favorable versus unfavorable functional outcomes would greatly enhance shared decision-making. Second, randomized trials comparing decompressive hemicraniectomy to newer medical therapies such as targeted temperature management or novel osmotic agents are needed to define the evolving role of surgery in the context of advancing medical therapies. Third, long-term follow-up studies examining quality of life, cognitive function, and caregiver burden beyond the first year post-stroke would provide a more complete picture of outcomes that matter to patients and families. Fourth, studies exploring the optimal timing of cranioplasty following decompressive hemicraniectomy and its impact on neurological recovery are needed to optimize the full surgical pathway (35).

## 5 Conclusion

In conclusion, this meta-analysis confirms that decompressive hemicraniectomy significantly reduces mortality in patients with malignant middle cerebral artery infarction while providing meaningful improvements in functional outcomes for many survivors. The survival benefit is sustained across all age groups and time points, and surgery does not increase the proportion of patients surviving with severe disability. The decision to recommend decompressive hemicraniectomy in the Neuro-ICU should be guided by a careful assessment of patient factors, with particular attention to age and pre-stroke functional status, and should be accompanied by comprehensive discussions that address both the survival benefit and the spectrum of possible functional outcomes. When performed in appropriately selected patients and supported by high-quality Neuro-ICU care, decompressive hemicraniectomy represents a life-saving salvage therapy that can achieve outcomes aligned with patient-centered goals of care (50).

## Data Availability

The original contributions presented in the study are included in the article/supplementary material, further inquiries can be directed to the corresponding author.
